# Supplementary material for: Optimal diversification strategies in the networks of related products and of related research areas
Source: Nat Commun. 2018 Apr 6;9:1328. doi: 10.1038/s41467-018-03740-9 (PMC5889401; doi:10.1038/s41467-018-03740-9)
Supplement: Supplementary file 1 — Supplementary Information(PDF 1515 kb) [file 41467_2018_3740_MOESM1_ESM.pdf]

Supplementary Material
Optimal diversification strategies in the networks of related products and of related research areas

Aamena Alshamsi[1,2,*], Flávio L. Pinheiro[1,*], and César A. Hidalgo[1]

[1]The MIT Media Lab, Massachusetts Institute of Technology, Cambridge, MA, USA
[2]Khalifa University of Science and Technology, Masdar Institute, Abu Dhabi, UAE
[*]These authors contributed equally to this work

Supplementary Note 1: Strategies

In this section, we discuss the properties of the main strategies explored along the manuscript. Each pure strategy is a simple heuristic that dictates how a player selects the next targeted node in a network for activation. We consider the following strategies,

S_1– **Random**, where nodes are selected at random for activation, this strategy forms a baseline scenario;

S_2– **High Degree**, where at each step, the node with the highest degree is selected for activation;

S_3– **Low Degree**, where at each step the node with the lowest degree is selected for activation;

S_4– **Greedy**, where at each step, the node with the highest probability of activation, and respectively low expected activation time is selected;

S_5– **Majority**, where at each step, the node with the highest number of connections to active nodes is selected.

These Strategies are graphically represented in Supplementary Figure 1 for a network with $Z = 58$ nodes of which three are active and four are potential, that is, have non-zero probability of Activation.

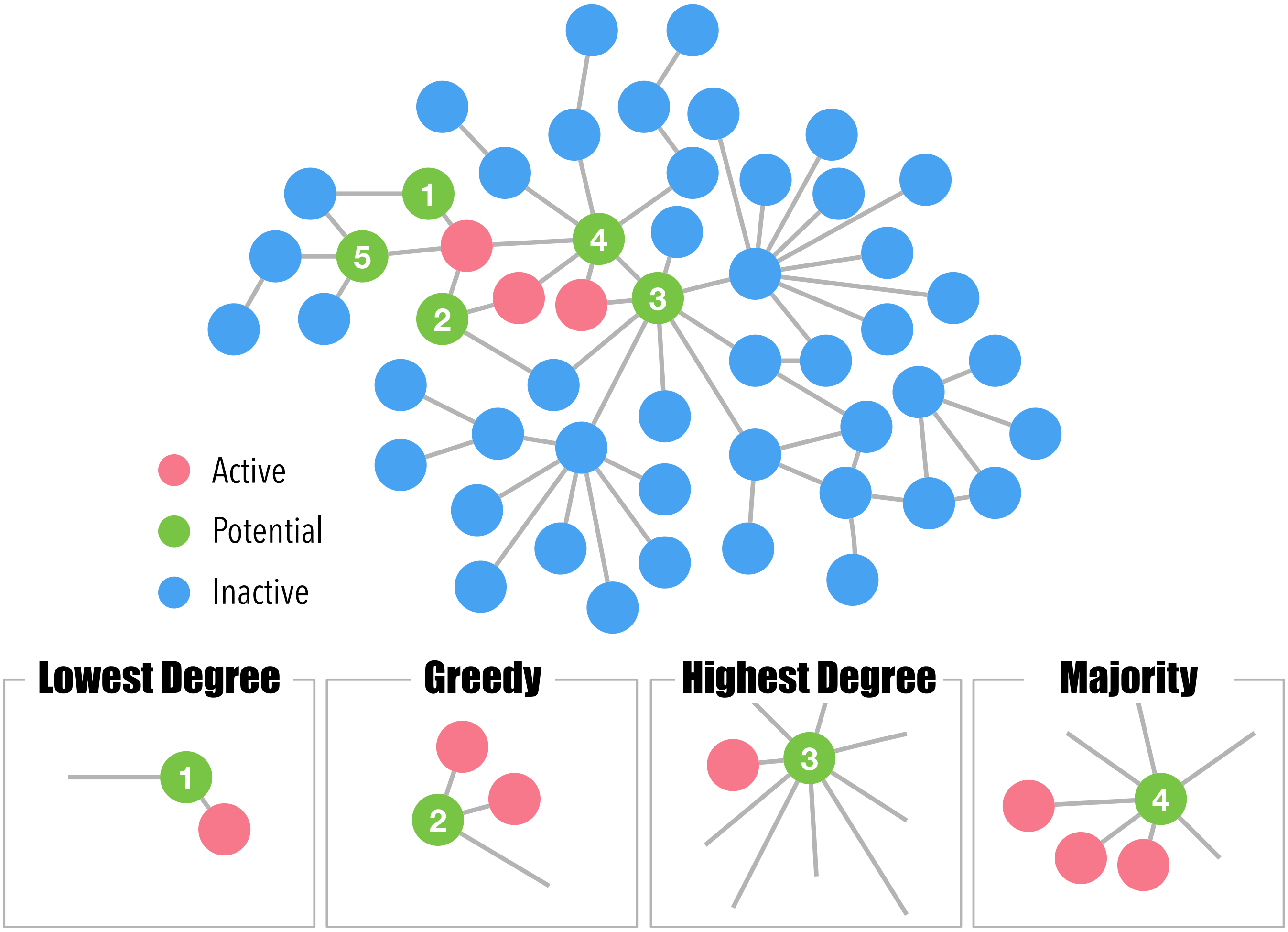

Supplementary Figure 1: Graphical depiction of different targeted nodes by different strategies in a network with $Z = 58$ nodes, of which three are Active (red) and four nodes have nonzero probability of activation (Green). The low-degree strategy would target node 1 which has a degree of two. The greedy strategy would target node 2 that has two active neighbors out of three. The high-degree strategy would pick node 3 which has a degree of nine. The majority strategy would pick node 4 that has three active neighbors. Additionally, the Random strategy would target any of the nodes with equal probability.

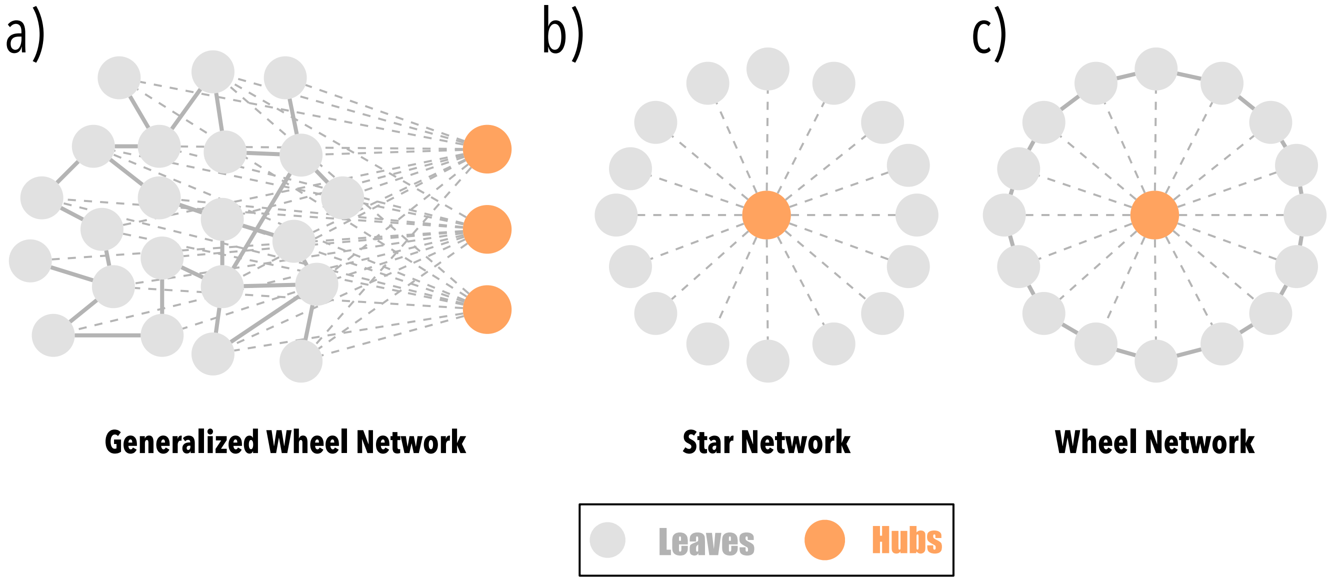

Supplementary Figure 2: Depiction of the Generalized Wheel Networks analyzed in this document and two limiting cases. a) **Generalized Wheel Network**–these structures are made of n leaves that form a random uncorrelated graph (full lines) with degree distribution $D(k)$, leaves are also randomly connected with hubs (dashed lines). b) **Star Network** – this network is a particular case of the **Generalized Wheel Network** in which the $Z-1$ leaves are connected to a single hub and are not connected among themselves. c) **Wheel Network** – this network is another particular case of **Generalized Wheel Network** made up of $Z-1$ leaves that are all connected to a single central hub and each is connected with two other leaves but do not form triangles.

These strategies can be combined to produce a more complete and rich set of strategies. The problem is, then, how to mix these strategies? A simple approach is to consider that strategies, in general, are but a linear combination of the five pure strategies listed above. In that sense, consider that the strategy is described by a vector $\vec{S} : \{\beta_1, \beta_2, \beta_3, \beta_4, \beta_5\}$ where β_i corresponds to the relative weight of strategy S_i and with $\sum_{i=1}^{5} \beta_i = 1.0$. Hence, strategies form a 4-Simplex space and β_i can be associated with the probability of playing a given strategy at each time step.

Supplementary Note 2: Network Structures

Here, we discuss the artificial structures that have been analyzed in the main text. This includes a family of structures that we denominate by *Generalized Wheel Networks* and the Scale Free networks generated through the configuration model [1].

Generalized Wheel Networks

A *Generalized Wheel Network* is composed by n *leaves* (peripheral nodes that have low degrees) and m *hubs*. The composition of the network is such that $Z = n+m$ and there are many more leaves than hubs ($n >> m$). Leaves form an uncorrelated random graph among themselves, which is characterized by a degree distribution $D(k)$ and average degree $\langle k_{\mathrm{L}} \rangle$. Each leaf can also be, or not, connected to one or more hubs. Therefore, hubs have larger effective degrees. Connections between leaves and hubs are formed at random. For simplicity we consider that hubs do not form connections between themselves and that all have the same degree of k_{H}. We also consider that the degree of a hub is much larger than the expected degree of a random leaf $k_{\mathrm{H}} >> \langle k_{\mathrm{L}} \rangle$. Leftmost graph in Supplementary Figure 2 depicts the topological properties of these structures.

These structures are simple enough to allow the inspection of analytical solutions while providing topological features that are present in real world structures with more complex connectivity profiles. Thus providing the perfect scenario for developing some intuition on the nature of optimal solutions.

Two limiting cases return familiar networks. When $m = 1$; $\langle k_{\mathrm{L}} \rangle = 0$ (leaves are not connected to each other) and $k_{\mathrm{H}} = n$ we obtain a *star network*, a structure dominated by a single hub fully connected to a set $Z-1$ of unconnected

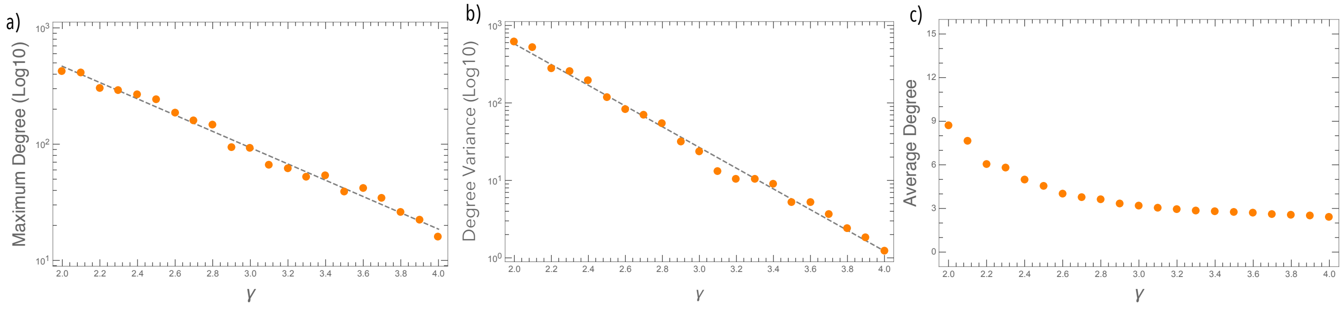

Supplementary Figure 3: Topological Properties of the Scale-Free Networks Generated with the Configuration Model. The panels show the expected maximum degree (a), the expected degree variance (b) and the expected average degree (c) as function of γ, the degree distribution power $D(k)\ k^{-\gamma}$. In a and b, the Y-Axis is in logarithmic scale of base 10.

Leaves (see Supplementary Figure 2 b). When $m = 1$; $\langle k_{\rm L} \rangle = 2$; $k_{\rm H} = n$ and in the absence of cliques (triangles) among the leaves we obtain a *wheel network* (see Supplementary Figure 2 c). In this work we explore the particular case of the *wheel networks* and *generalized wheel Networks*.

Scale-Free Networks

In order to approach more realistic structures we analyze the performance of different strategies on uncorrelated scale-free networks. These structures are characterized by a power-law degree distribution $(D(k) \approx k^{-\gamma}$ and have been generated using the configuration model [1].

We have generated 100 independent networks with $Z = 10^3$ nodes for a total of 20 different values of γ equally distributed between $\gamma = 2.0$ and $\gamma = 4.0$. Supplementary Figure 3 shows the main topological features – Maximum Degree, Degree Variance and Average Degree – of the networks under analysis as a function of γ.

Supplementary Note 3: Diffusion Time using Sequential Targeting

A simple strategy, inspired by the topology of *Wheel Networks*, involves the sequential activation of *leaves* and *hubs*. That is, we start by activating L leaves, followed by the activation of all m hubs before activating the remaining $(n - l)$ leaves. Hence, the problem resumes to find the optimal l^* that minimizes the total diffusion time.

In *wheel networks* this problem can be broken down in to the computation of the diffusion time in three independent parts, that is,

$$T(L,\alpha) = \sum_{i=2}^{l} \tau_{\rm leaf}(i,0,\alpha) + \sum_{i=1}^{m} \tau_{\rm hub}(L,i,\alpha) + \sum_{i=l+1}^{n} \tau_{\rm leaf}(i,m,\alpha) \quad (1)$$

where the term $\tau_{\rm leaf}(X,Y,\alpha)$ corresponds to the activation time of the $X^{\rm th}$ leaf when there are Y active hubs and $X - 1$ active leaves. Likewise $\tau_{\rm hub}(X,Y,\alpha)$ corresponds to the activation time of the $Y^{\rm th}$ hub when there are X active Leaves and $Y - 1$ active hubs. Hence, the first sum goes over the activation of the first L leaves, the second sums the activation of the hubs and the third over the activation of the remaining $n - L$. A natural limitation of this strategy is that it can only be applied to networks that exhibit a strong variance in the degree distribution. In other words, when $k_{\rm H} >> k_{\rm L}$. In the following sections we show the solution for *wheel networks* and an approximation for *generalized wheel networks* according to this sequential strategy.

The Wheel Network

In the case of a wheel network $m = 1$ and $n = Z - 1$. Assuming that we start with a single active leaf, the activation time of the first $L - 1$ leaves is

$$\sum_{i=2}^{L} \tau_{\rm leaf}(i,0,\alpha) = (L-1)\left(\frac{1}{3}\right)^{-\alpha} \quad (2)$$

the activation of the hub is

$$\tau_{\rm leaf}(L,1,\alpha) = \left(\frac{L}{n}\right)^{-\alpha} \quad (3)$$

and, finally, the activation of the remaining leaves is

$$\sum_{i=L+1}^{n_{\rm L}} \tau_{\rm leaf}(i,1) = (n-l-1)\left(\frac{2}{3}\right)^{-\alpha} + 1 \tag{4}$$

where the addition of 1 accounts for the activation time of the last leaf, which is surrounded by three active neighbours. Hence, the total activation time of the entire wheel network becomes

$$T(L,\alpha) = (L-1)\left(\frac{1}{3}\right)^{-\alpha} + \left(\frac{L}{n}\right)^{-\alpha} + (n-L-1)\left(\frac{2}{3}\right)^{-\alpha} + 1 \tag{5}$$

Since the goal is to find how many leaves should be activated before activating the hub we proceed by finding the derivative of τ with respect to l, which is

$$\frac{dT(L,\alpha)}{dL} = -\left(\frac{3}{2}\right)^{\alpha} + 3^{\alpha} - \alpha\left(\frac{L}{Z-1}\right)^{-(\alpha+1)}\frac{1}{Z-1} \tag{6}$$

and solving $d\tau/dl = 0$ in order to l we find that

$$L^* = (Z-1)\left(\frac{((3/2)^{\alpha} + 3^{\alpha})(Z-1)}{\alpha}\right)^{-\frac{1}{\alpha+1}} \tag{7}$$

An interesting result, which can be derived from this solution, is that the optimal step (L^*) to activate the hub is always lower than the moment in which the probability of activating the hub becomes larger than the probability to activate the leaves, that is $L^* < Z/3$, which would be the approach adopted by a greedy strategist.

Panel **A** of Supplementary Figure 4 shows the activation time for $\alpha = 1.5$ for different network sizes (Z) while panel **B** shows how the solution, L^*, varies for different values of $\alpha \in]0,2]$ and different network sizes (Z).

Generalized Wheel Networks

After solving for the wheel network it becomes conceptually easy to solve for the *generalized wheel networks*, of which the Wheel network is a particular case. Let's assume that the leaves form an uncorrelated random graph with degree distribution $D(k)$ and average degree $\langle k\rangle$. The expected effective degree of each leaf ($k'_{\rm leaf}$), after accounting for the potential connections with the hubs, is

$$k'_{\rm leaves} = k_{\rm leaf} + \frac{m k_{\rm leaves}}{n} \tag{8}$$

We approximate the first part of Equation by

$$\sum_{i=2}^{l} \tau_{\rm leaf}(i,0,\alpha) = \sum_{j=2}^{L}\sum_{i=0}^{k'_{\rm leaf}-1} \binom{n-2}{3}^{-1}\binom{j-1}{k}\binom{n-j-1}{3-k}\left(\frac{k'_{\rm leaf}}{(1+i)}\right)^{\alpha} \tag{9}$$

The first sum adds up the expected activation time of the first l nodes. Each target leaf node is connected to at least one active node, so the second sum goes over how many more active leaves might be connected to it, thus goes from $i = 0$ to $k_{\rm leaf} - 1$. Each possibility is weighted using the hypergeometric distribution, which computes the probability of sampling i active nodes and $k_{\rm leaf} - i$ inactive from a population with $j - 1$ active and $n - j - 1$ inactive nodes.

In the limit that $n \to \infty$ and upon the change $n = 1/Y$, Taylor expanding in respect to Y leads to

$$\sum_{i=2}^{L} \tau_{\rm leaf}(i,0,\alpha) \approx \sum_{j=1}^{L}\left[k_{\rm leaf}^{\alpha} - \left(\frac{3}{2}(j-1)k_{\rm leaf} + k_{\rm hub}m\right)Y + O(Y^2)\right] \tag{10}$$

revealing that in the limit of $n \to \infty$ the first part of Equation 3 reduces to $\sum_{i=2}^{L}\tau_{\rm leaf}(i,0,\alpha) \approx k_{\rm leaf}^{\alpha}$ if we drop terms proportional to $1/n$.

The second part of Equation 3 corresponds to the time it takes to activate m hubs having L active leaves, which can be computed as

$$\sum_{i=1}^{m} \tau_{\rm hub}(L,i,\alpha) = m\sum_{i=0}^{L-1}\binom{L}{i}\binom{n-L-1}{k_{\rm hub}-i}\binom{n}{k_{\rm hub}}\left(\frac{k_{\rm hub}}{i+1}\right)^{\alpha} \tag{11}$$

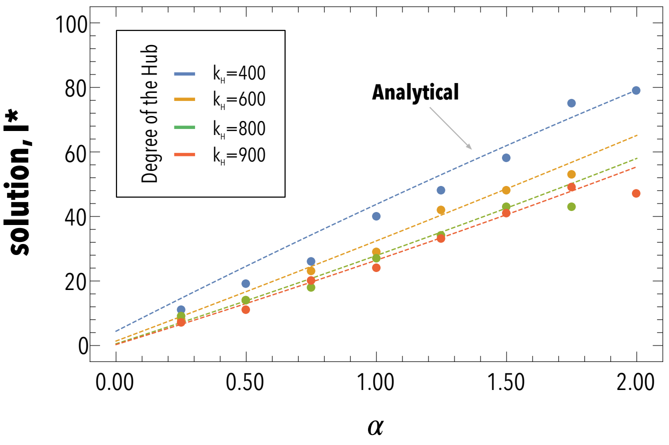

Supplementary Figure 4: Solutions for the generalized wheel network using a sequential strategy of activation. Solutions (L^*) for structures with different densities of links between hubs and leaves as a function of the parameter α. Analytical predictions correspond to the dashed lines while dots are numerically estimated solutions. Networks have $Z = 10^3$ nodes, $m = 2$ hubs and $n = 998$ leaves.

since hubs are not connected, the expected time of activation of each hub is independent. hubs are connected to at least one active leaf and the sum goes over the possibility of the hub to be connected with up to L leaves.

The final part of Equation 3, follows a similar form as Equation 9, but sums the activation times of the remaining $n - L - 1$ Leaves, that is,

$$\sum_{i=L+1}^{n} \tau_{\text{leaf}}(i, m, \alpha) = \sum_{j=L+1}^{n} \sum_{k=0}^{3} \binom{n-1}{4}^{-1} \binom{j}{k} \binom{n-j-1}{4-k} \left(\frac{mk_{\text{hub}} + nk_{\text{leaf}}}{mk_{\text{hub}} + kn} \right)^{\alpha} \tag{12}$$

a small modification to the last factor is introduced in order to account for the k_{hub} that are now active. Supplementary Figure 4 shows the predicted solutions from the analytical model (dashed) versus the solutions obtained numerically for networks with different properties as a function of α.

The analytical result agrees with the simulations for a large range of parameters, in particular for large k_{hub} and for increasing m. The analytical result agrees with simulations in the case where the number of connections between the hubs and leaves is sufficiently large so that all hubs are connected to at least one leaf.

For networks in which hubs and leaves are weakly connected the analytical approximation fails since it doesn't account for the case in which a component of leaves that reach L is not connected to any of the hubs.

Supplementary Note 4: Optimal time to target heterogeneous networks

In this section we extend the results on heterogeneous networks for a wider range of parameters. In particular we show that what makes the mixed strategy an optimal strategy is the ability to target higher degree nodes (hubs) at the right time for a wide range of networks. A timing that other strategies tend to overestimate (Greedy and Low Degree) or underestimate (Majority and High Degree).

Supplementary Figure 5 shows a color map representing the probability that hubs are activated at a particular time of the activation process (Y-axis) for a wide range of networks exhibiting different levels of degree heterogeneity (X-axis). Darker areas represent higher probabilities and lighter areas represent lower probabilities. the top 15% most connected nodes as hubs, for each network. Moreover, we take only the time series in which the diffusion time is lower than the average of a sample constituted by 10^5 independent time series. This selection allows us to exclude examples of time series whose diffusion time is affected by a poor initial condition.

Supplementary Figure 5 a, b and c show the results for $\alpha = 1.0$ and Supplementary Figure 5 d, e and f show the results for $\alpha = 2.0$. As expected, the Majority strategies remain the same pattern of activation regardless of the value of α, that is, the difficulty of the diffusion process. The greedy strategy targets hubs latter with increasing γ, that is, decreasing heterogeneity of the network. The optimal mixed strategy is however able to adapt and target hubs at the right time depending of the context, both α and the level of heterogeneity of the network.

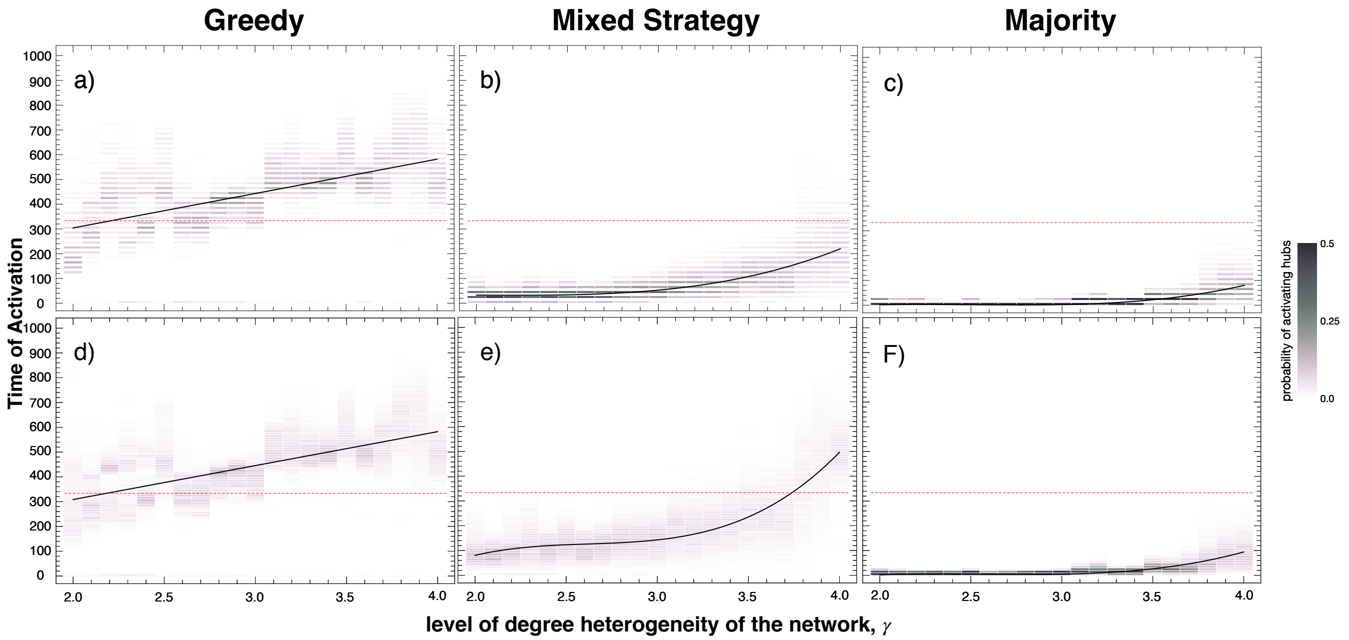

Supplementary Figure 5: Time at which each strategy targets the most well connected nodes – hubs – for a wide range of heterogeneous networks (X-axis) when $\alpha = 1.0$ in the top panels and $\alpha = 2.0$ in the bottom panels. The Y-axis represents the position at which nodes can be activated. Each panel concerns a different strategy. Darker colors represent higher probability of a hub being activated at that stage. Lines show a polynomial regression over the expected values obtained for each network. Networks have $Z = 10^3$ nodes and have been generated using the configuration model with a degree distribution that follows a power law $\approx k^{-\gamma}$.

Supplementary Note 5: Product Space

The Product Space is a weighted network that represents the relationship between products according to the likelihood they are co-exported by the same country [2]. The products that are co-exported more often have stronger relationships (higher proximity), while products that are rarely co-exported together have weaker relationships indicating they are distant from each other. This is manifested by the network structure of the product space. Next, we briefly describe the data source used to generate the product space and afterwards we show how the product space can be estimated.

We use the dataset of the MIT's Observatory of Economic Complexity (atlas.media.mit.edu) [3]. This data set combines exports data from 1962 to 2000, compiled by Feenstra et al. [4], and data from the U.N. Comtrade for the period between 2001 and 2012. As is common practice in the literature of Economic Complexity and the Product Space [9] we exclude countries with less than 1.2 million citizens and with total trade below USD 1 billion in 2008. Also ountries with poor data on exports such as Iraq, Chad and Macau are excluded. The sample of the data accounts for 99% of World trade, 97% of World total GDP and 95% of World population.

We have obtained data about countries' population from the World Bank's Worldwide Indicators (http://data.worldbank.org). For more information, check [2].

A country is said to have developed a product when it exhibits Revealed Comparative Advantage (RCA) in that product, which can be computed in two ways. The first compares the share of the exports of a product in the total exports of a country with the share of exports that product in relation to the total volume of exports of the world, that is

$$\mathbf{RCA}_{cp} = \frac{X_{cp}}{\sum_{cp'} X_{cp'}} \Bigg/ \frac{\sum_{c'p} X_{c'p}}{\sum_{c'p'} X_{c'p'}} \tag{13}$$

where X_{cp} is the total exports of product p of country c. When the **RCA** is larger than 1 (indicating that a country has *comparative advantage* in a product) the share of exports of that product in a country's economy is larger than the share of exports of that product in the global market.

The second approach compares the exports *per capita* of a product in a country with the exports per capita of that product in the world [5]. In this case, X_{cp} in Equation 14 is the total export of product p by country c *per*

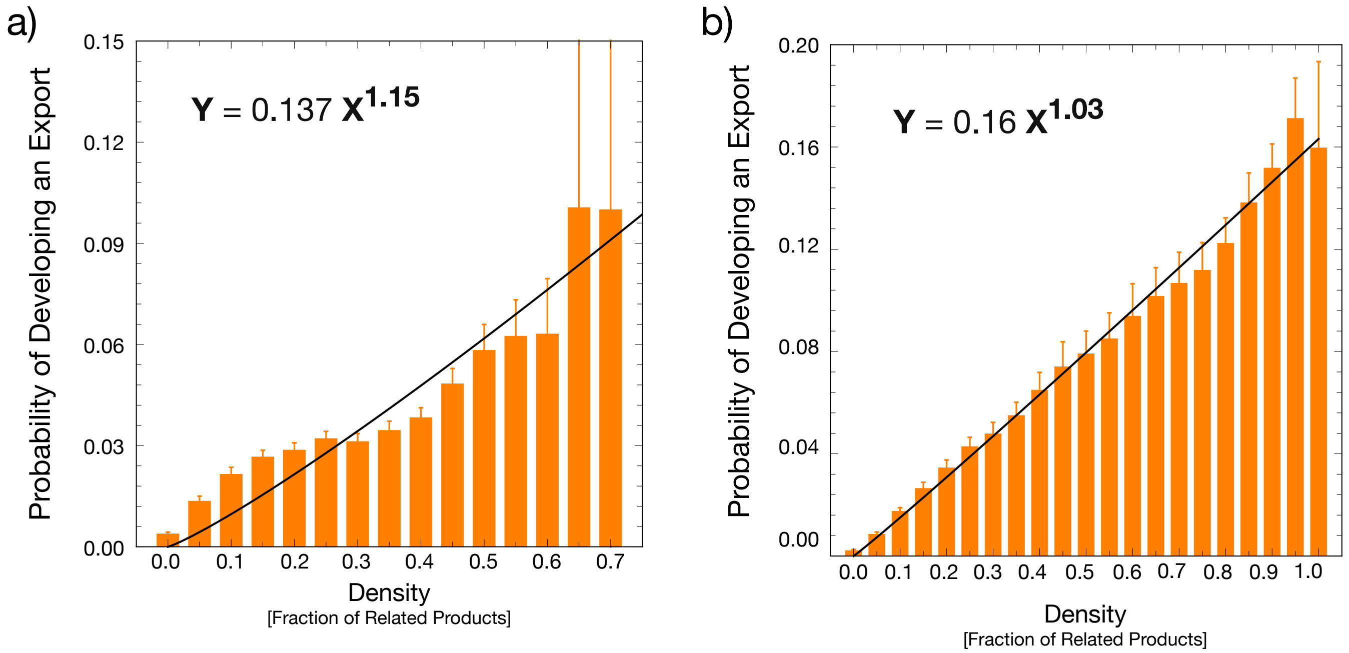

Supplementary Figure 6: Probability of developing a new export in the Product Space as a function of the density of developed exports in the vicinity of a product. a) shows the relationship obtained in the original Product Space. b) shows the relationship obtained after simplifying the Product Space from a weighted to an unweighted network as described in the text. In both cases we obtain a relationship that shares the qualitative properties, namely a convex profile with $\alpha > 1.0$ indicating the existence of a complex contagion process.

capita and is as follows

$$\mathbf{RCA}_{cp}^{cap} = \frac{X_{cp}}{N_c} \Bigg/ \frac{\sum_{c'p} X_{c'p}}{\sum_{c'} N_{c'}} \tag{14}$$

where N_c represents the population of country c and $\mathbf{RCA}_{cp}^{cap}$ is the Relative Comparative Advantage *per capita*. In this case, $\mathbf{RCA}_{cp}^{cap}$ larger than 1 implies that the exports *per capita* of the product by a country are larger than the exports *per capita* of that same product in the world. This, however, has proven to be a very stringent criteria and often the threshold is set to 0.25 [5, 6]

By using RCA, it is possible to construct a matrix, M_{cp}, whose entries are 1 if country c has $\mathbf{RCA}> 1$ in product p and 0 otherwise, that is

$$M_{cp} = \begin{cases} 1 & \mathbf{RCA}_{cp} \geq 1 \\ 0 & \mathbf{RCA}_{cp} < 0 \end{cases} \tag{15}$$

which represents the adjacency matrix of a bipartite network connecting countries and products. From this structure we can estimate the proximity between every pair of products, $\phi_{pp'}$, as the conditional probability that a country has **RCA** in both given that it has **RCA** in one of them [7]. This is formulated as

$$\phi_{pp'} = \frac{\sum_c M_{cp} M_{cp'}}{max(k_{p0}, k_{p'0})} \tag{16}$$

which means, for instance, that if two products have a proximity of 0.4 then there is a 40% chance that a random country with **RCA** in one product has **RCA** in both.

The resulting network, the so called Product Space, is thus a weighted graph whose weights correspond to the conditional probabilities of the two products being co-exported by the same country. This contrasts with the unweighted structures explored in the main manuscript. To that end we build a simplified version of the Product Space in which we discard all edges that have a weight lower than a specified cutoff value – here defined as the lowest possible weight necessary to obtain a single giant component – and convert the weight of the remaining edges to 1.

We consider that, for each country, a product is active if the country has an RCA *per capita* greater than 0.25 over such product [5, 6]. To capture the development strategies of countries we look for products which undergo a transition in the RCA from lower than 0.25 to an RCA greater than 0.25 in consecutive years (e.g. from y to

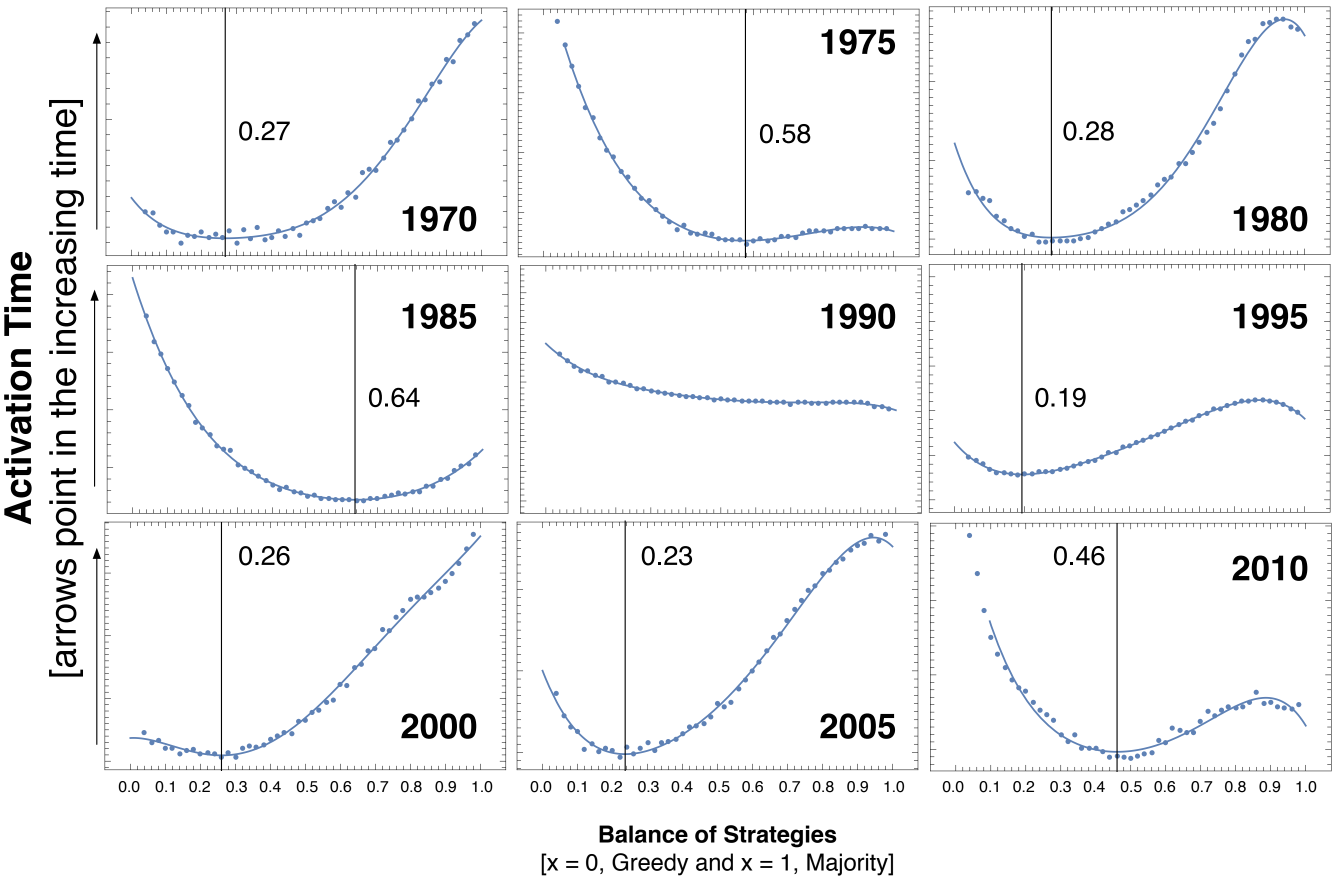

Supplementary Figure 7: In each panel we show the activation times (Y-Axis) as a function of a balance of Greedy and Majority actions in a mixed strategy profile (X-Axis). When $x = 0$ the activation follows the Greedy strategy, while when $x = 1$ the activation follows the Majority activation strategy. Each panel, corresponds to the simplified product space for different years from 1970 to 2010. In all, except one panel, there is always an optimal balance of actions that minimizes the activation time. Without loss of generality, in the main manuscript we use the network obtained from the product space in 2005.

$y+1$) We do such analysis between the years of 1970 and 2010. For each product that underwent such transition we compute the degree and density of active products in its vicinity. This way we can compute the probability that a country develops a product as a function of the density of active products in its vicinity. We use this information to build Figure 1c of the main manuscript.

We validate the simplified Product Space by comparing the probabilities of development of new products between two consecutive years obtained from both the simplified and the original Product Space (weighted network), see Supplementary Figure 6. The results show that both structures capture the fundamental dynamical aspect of complex contagion and qualitatively exhibit a super-linear relationship.

Moreover, in Supplementary Figure 7 shows the optimal activation strategy for the simplified Product Space in different years. In all cases, except for 1990, the optimal strategy (the one that minimizes the activation time, always mixes between Greedy and Majority strategies. In the main manuscript we use, without loss of generality, the product space in 2005.

Supplementary Note 6: Research Space

The research space is a weighted network that represents the relationship between research fields, measured by the likelihood that authors co-publish in two different fields [8]. Pairs of research fields that authors tend to co-publish in, more often are associated with stronger relationships (higher proximity), while research fields in which authors rarely co-publish in, have weaker relationships. This is manifested by the network structure of the research space. Next, we describe the data used to generate the Research Space and afterwards we discuss the building blocks of the

research space.

Following Guevera et al [8] we make use of a dataset containing 319,049 authors who have authored a total of 4,745,774 publications indexed in 16,873 journals and proceedings between 1971 and 2014. The data was obtained from Google Scholar and only authors with a verified account are considered, which allows us to overcome many problems with author name ambiguity. We note that the Google Scholar dataset is not free of biases, as the adoption of Google Scholar is not uniform across academic fields, or age groups. So we interpret our results in the narrow context of the data used to produce them.

Additionally, we filter this dataset by focusing only on scholars with less than fifty publications in each year, because those with more than fifty publications tend to have many publications that are miss-assigned and are not theirs (see [8] for more details). Our filtered dataset contains 319,049 authors who have authored a total of 4,745,774 publications indexed in 16,873 journals and proceedings between 1971 and 2014.

We assign each publication to a research category based on the journal in which it was published using Scopus classification system provided by SCImago that includes 27 main areas of knowledge that are subdivided into 308 fine grained categories. In our dataset we use only the 2 categories for which at least one paper was found.

The presence of scientist s in a research field f at time t ($X_{sf}(T)$) is calculated by taking the sum of all papers that are published by scientist s in the research field f before time T. Then, this quantity is normalized by the total number of the scientist's co-authors who co-produced each paper np and the number of research fields at which each paper is published mp. Then $X_{sf}(T)$ is discretized to discard scientists who did not significantly contributed to a given research field.

$$X_{sf}(T) = \sum_{p(s,f,T)} \frac{1}{n_{p(s,f,T)} m_{p(s,f,T))}} \tag{17}$$

$$P_{sf}(T) = \begin{cases} 1 & \mathbf{X}_{sf} \geq 0.1 \\ 0 & \mathbf{X}_{sf} < 0.1 \end{cases} \tag{18}$$

Then the we calculate the number of scientists who published in fields f and f' before time T by multiplying $P_{sf}(T)$ by itself as follows.

$$M_{ff'}(T) = \sum_s P_{sf}(T) P_{sf'}(T) \tag{19}$$

Then, the proximity between fields $\phi_{ff'}$ is calculated as follows.

$$\phi_{ff'}(T) = \frac{M_{ff'}}{\sum_s P_{sf'}} \tag{20}$$

$\phi_{ff'}(T)$ denotes the proximity between two fields and represents the weight of the link between f and f'. The resulting network, the so called Research Space, is thus a weighted and directed graph whose weights correspond to the proximity between research fields.

Again, this contrasts with the unweighted and undirected nature of the network structures explored in the main manuscript. To that end we build a simplified version of the Research Space: (i) we replace directed nodes by an undirected node whose weight is the average of the two initial links and (ii) we discard all edges that have a weight lower than a specified cutoff value – here defined as the lowest possible weight necessary to obtain a single giant component – and convert the weights of the remaining edges to 1.

We used data from 1971 to 2010 to construct the proximity matrix, which we used to both run our simulations and to study the diversification of countries research fields between 2008 and 2013. The smaller window of observation in the case of the Research space is due to the sparse sample of authors before 2008 that would result in an incomplete and poor representation of the developed research areas by country, see Guevera et al [8].

We consider that, for each country, a research field is active if the country has an RCA per capita greater than 1. To capture the development strategies of countries we look for which research fields undergo a transition in the RCA per capita from lower than 1 to an RCA per capita greater than 1 in consecutive years (e.g. from y to y + 1). We do such analysis between the years of 2008 and 2011. For each research field that underwent such transition we compute the degree and density of active research field in its vicinity. Then we can compute the probability that a country develops a research field as a function of the density of active products in its vicinity. We use this information to build Figure 1d of the main manuscript.

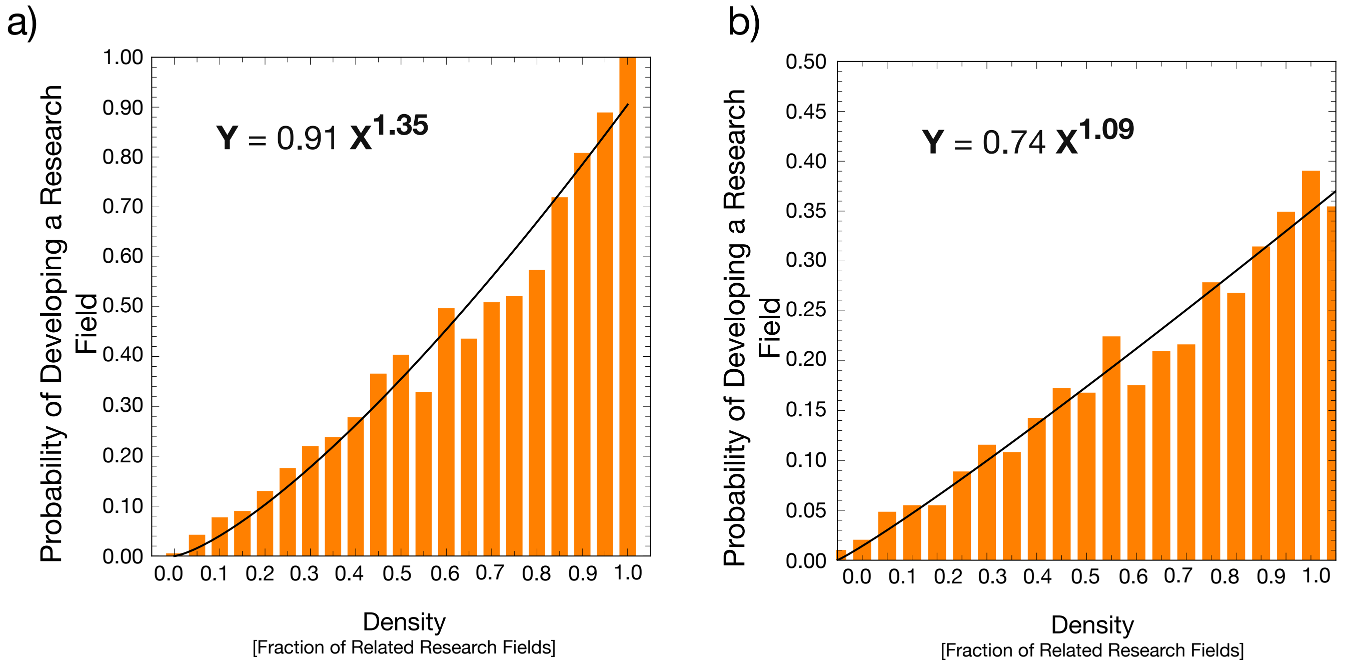

Supplementary Figure 8: Probability of developing a new research field in the Research Space as a function of the density of developed fields in the vicinity of an undeveloped research field . a) shows the relationship obtained in the original Research Space. b) shows the relationship obtained after simplifying the Research Space from a weighted to an unweighted network as described in the text. In both cases we obtain a relationship that shares the qualitative properties, namely a convex profile with $\alpha > 1.0$ indicating the existence of a complex contagion process.

Supplementary References

[1] Newman, M. E. The structure and function of complex networks. *SIAM review* **45**, 167–256 (2003) https://doi.org/10.1137/S003614450342480.

[2] Hidalgo, C. A., Klinger, B., Barabási, A.-L. & Hausmann, R. The product space conditions the development of nations. *Science* **317**, 482–487 (2007) https://doi.org/10.1126/science.1144581.

[3] Simoes, A. J. G. & Hidalgo, C. A. The economic complexity observatory: An analytical tool for understanding the dynamics of economic development. In *Scalable Integration of Analytics and Visualization* (2011).

[4] Feenstra, R. C., Lipsey, R. E., Deng, H., Ma, A. C. & Mo, H. World trade flows: 1962-2000. Tech. Rep., National Bureau of Economic Research (2005).

[5] Felipe, J. & Hidalgo, C. Economic diversification implications for kazakhstan. *Dev. Mod. Ind. Policy Pract.: Issues Ctry. Exp.* 160–196 (2015).

[6] Bustos, S., Gomez, C., Hausmann, R. & Hidalgo, C. A. The dynamics of nestedness predicts the evolution of industrial ecosystems. *PloS One* **7**, e49393 (2012) https://doi.org/10.1371/journal.pone.0049393.

[7] Hidalgo, C. A. & Hausmann, R. The building blocks of economic complexity. *Proc. Natl Acad. Sci. USA* **106**, 10570–10575 (2009) https://doi.org/10.1073/pnas.0900943106.

[8] Guevara, M. R., Hartmann, D., Aristarán, M., Mendoza, M. & Hidalgo, C. A. The research space: using career paths to predict the evolution of the research output of individuals, institutions, and nations. *Scientometrics* **109**, 1695–1709 (2016) https://doi.org/10.1007/s11192-016-2125-9.

[9] Bahar, D., Hausmann, R., & Hidalgo, C. A. Neighbors and the evolution of the comparative advantage of nations: Evidence of international knowledge diffusion? *Journal of International Economics* **92**, 111–123, (2014)
